# Urinary Metabolomic Alterations Associated with Contrasting Ambient Air Pollution Exposure in Thai Adults: An Untargeted ^1^H-NMR Study

**DOI:** 10.3390/ijms27167193

**Published:** 2026-08-12

**Authors:** Blecious Zinan’dala, Anupon Iadnut, Chikondi Maluwa, Puriwat Fakfum, Churdsak Jaikang, Giatgong Konguthaithip, Kanokwan Kulprachakarn, Wason Parklak, Hataichanok Chuljerm

**Affiliations:** 1 School of Health Sciences Research, Research Institute for Health Sciences, Chiang Mai University, Chiang Mai 50200, Thailand; dysonblecious_zina@cmu.ac.th (B.Z.); chikondi_maluwa@cmu.ac.th (C.M.); puriwat_f@cmu.ac.th (P.F.); kanokwan.kul@cmu.ac.th (K.K.); wason.p@cmu.ac.th (W.P.); 2 Faculty of Clinical Sciences, Malawi College of Health Sciences, Lilongwe 3, Lilongwe P.O. Box 30368, Malawi; 3 Research Institute for Health Sciences, Chiang Mai University, Chiang Mai 50200, Thailand; anupon_iad@nation.ac.th; 4 Faculty of Medicine, Nation University, Chiang Mai 50210, Thailand; 5 Department of Forensic Medicine, Faculty of Medicine, Chiang Mai University, Chiang Mai 50200, Thailand; churdsak.j@cmu.ac.th (C.J.); kongkiat.shang@gmail.com (G.K.); 6 Metabolomic Research Group for Forensic Medicine and Toxicology, Department of Forensic Medicine, Faculty of Medicine, Chiang Mai University, Chiang Mai 50200, Thailand

**Keywords:** air pollution, ambient PM_2.5_, urinary metabolomics, ^1^H-NMR, metabolic profiling, environmental exposure

## Abstract

Ambient fine particulate matter (PM_2.5_) is associated with oxidative stress, metabolic dysregulation, and cardiometabolic disease. However, systemic metabolic responses to contrasting real-world ambient air pollution exposure environments remain poorly characterized. In this cross-sectional study, untargeted proton nuclear magnetic resonance (^1^H-NMR)-based urinary metabolomics was used to investigate metabolic signatures associated with contrasting ambient air pollution exposure environments in Thailand. Adults residing in Chiang Mai (high-exposure region; n = 51) and Songkhla (low-exposure region; n = 52) were recruited during a period of elevated regional air pollution, with long-term residence serving as a proxy for differential exposure to ambient air pollution environments. Partial least squares-discriminant analysis (PLS-DA) demonstrated separation between exposure groups (R^2^ = 0.873, Q^2^ = 0.447), indicating good model fit but only modest predictive ability. Eight urinary metabolites differed significantly (*p* < 0.05), implicating pathways related to tryptophan metabolism, nucleotide metabolism, energy metabolism, and host–microbial co-metabolism. L-arginine and L-cystathionine showed lower relative abundance in the high-exposure group, and six metabolites remained significant after Benjamini–Hochberg false discovery rate correction. Following covariate adjustment, L-tryptophan, hippuric acid, xanthine, and 5-hydroxyindoleacetic acid (5-HIAA) remained significantly associated with the high-exposure group. Contrasting ambient air pollution exposure environments, indexed by regional PM_2.5_ concentrations, were associated with coordinated urinary metabolic alterations. Because long-term regional residence served as a proxy for individual-level exposure and diet and lifestyle differences between regions were not fully controlled, these findings should be interpreted as associations with contrasting regional exposure environments. Metabolite annotations remain putative and require validation in longitudinal studies with comprehensive pollutant characterization and individual-level exposure assessment.

## 1. Introduction

Fine particulate matter with an aerodynamic diameter ≤ 2.5 µm (PM_2.5_) represents one of the most pervasive environmental health threats globally, constituting a leading contributor to the burden of non-communicable diseases (NCDs), particularly cardiometabolic disorders [1]. Long-term exposure to PM_2.5_ has been consistently associated with increased cardiovascular morbidity and mortality [2]. Due to their submicron size, PM_2.5_ particles can penetrate deep into the pulmonary alveoli and subsequently translocate into systemic circulation, where they can induce oxidative stress, chronic inflammation, endothelial dysfunction, mitochondrial impairment, and metabolic disturbances that collectively contribute to cardiometabolic disease [3,4].

The health effects of PM_2.5_ are influenced not only by particle mass concentration but also by its chemical composition, which varies substantially according to pollution source, geographic location, and seasonal conditions [5]. These variations may lead to differences in biological responses across populations exposed to distinct pollution mixtures. Among the components of PM_2.5_, combustion-related compounds, including polycyclic aromatic hydrocarbons (PAHs), transition metals, and other redox-active compounds, are recognized contributors to oxidative stress and inflammation, although their relative roles may vary depending on the overall composition of the particulate mixture [6,7]. Consequently, understanding area-specific health risks requires not only monitoring ambient PM_2.5_ mass concentrations but also characterizing associated biological responses in exposed populations.

Thailand provides a unique setting for investigating PM_2.5_-related health effects due to pronounced geographic and seasonal contrasts in exposure. Northern Thailand, particularly Chiang Mai province, experiences recurrent episodes of markedly elevated PM_2.5_ during the dry season (February to May), driven by a combination of seasonal biomass burning, agricultural activities, and traffic-related emissions [8]. According to data from the Pollution Control Department (PCD) monitoring stations of Thailand, 24 h average PM_2.5_ concentrations during haze episodes in Chiang Mai can exceed 150 µg/m^3^, substantially exceeding the World Health Organization (WHO) 24 h guideline of 15 µg/m^3^ and the Thai national standard of 37.5 µg/m^3^. In contrast, 24 h average PM_2.5_ concentrations in southern coastal regions such as Songkhla are generally considerably lower than those in Chiang Mai during the same period, reflecting the influence of maritime airflow and higher precipitation [8,9]. These contrasting ambient air pollution exposure environments provide a valuable real-world framework for examining biological responses associated with long-term differences in ambient air pollution exposure.

Increasing evidence indicates that ambient air pollution acts as a systemic metabolic stressor capable of perturbing multiple interconnected biochemical pathways relevant to cardiovascular and metabolic health [10]. Oxidative stress and inflammation induced by PM_2.5_ exposure have been associated with disturbances in amino acid metabolism, mitochondrial energy production, purine and pyrimidine metabolism, nitric oxide signaling, sulfur amino acid metabolism, and gut microbial co-metabolism [11,12,13,14,15,16]. These metabolic alterations are closely linked to redox homeostasis, endothelial function, immune regulation, and cellular energy balance. For example, inflammatory activation alters tryptophan metabolism through the kynurenine pathway, while oxidative stress promotes purine degradation via xanthine oxidoreductase activity and influences redox-sensitive pathways involved in nitric oxide production and transsulfuration [11,12,13,14,15,16,17,18,19]. Collectively, these mechanistic studies provide biological plausibility that long-term ambient air pollution exposure may be reflected by coordinated alterations across interconnected metabolic pathways rather than isolated changes in individual metabolites. Accordingly, comprehensive metabolomic profiling provides an opportunity to characterize these coordinated biochemical responses and to identify metabolic signatures associated with environmental exposure.

Untargeted metabolomics offers a comprehensive approach for characterizing systemic metabolic responses to environmental exposures and identifying early biochemical perturbations relevant to disease risk [20,21]. Among metabolomics platforms, proton nuclear magnetic resonance (^1^H-NMR) spectroscopy provides reproducible and non-destructive metabolic profiling with minimal sample preparation, making it suitable for exploratory population-based studies [22]. Metabolomics has increasingly been applied in environmental health research to characterize biological responses to complex environmental exposures and to identify metabolic pathways associated with oxidative stress, inflammation, energy metabolism, and gut microbial co-metabolism, thereby providing mechanistic insight beyond conventional exposure assessment [23,24,25]. Urine is a particularly suitable, non-invasive biofluid for this purpose, as it reflects a wide range of endogenous metabolic processes and excreted compounds associated with physiological and environmental influences [21]. Because urinary metabolites are influenced by multiple factors, including environmental exposures, diet, gut microbial metabolism, and host physiology, interpretation of individual metabolite alterations requires consideration of the broader biological and exposure context. Previous studies have applied urinary metabolomics to investigate metabolic alterations associated with air pollution exposure [26,27,28]. Despite these advances, population-based studies examining urinary metabolomic profiles associated with contrasting long-term ambient air pollution exposure environments remain limited, particularly in Southeast Asian populations, where emission sources, climatic conditions, and PM_2.5_ composition differ substantially from those reported elsewhere.

To address this knowledge gap, we applied untargeted proton nuclear magnetic resonance (^1^H-NMR)-based urinary metabolomics to characterize metabolic signatures associated with contrasting ambient air pollution exposure environments in two regions of Thailand with markedly different ambient PM_2.5_ concentrations, as documented by the Thailand PCD database. Differential urinary metabolites and associated metabolic pathways were identified to improve understanding of biological responses associated with contrasting long-term ambient air pollution exposure environments. We hypothesized that individuals residing in the higher-exposure region would exhibit distinct urinary metabolic profiles, particularly in metabolic pathways related to oxidative stress and energy metabolism.

## 2. Results

### 2.1. Demographic Characteristics

The demographic and baseline characteristics of study participants are summarized in Table 1. A total of 103 participants were enrolled (Chiang Mai, n = 51; Songkhla, n = 52). Overall, baseline characteristics were comparable between groups, with no significant differences observed in age, sex, body mass index (BMI), blood pressure, heart rate, smoking status, or occupational distribution. However, the proportion of participants reporting alcohol consumption was significantly higher in Chiang Mai compared with Songkhla (*p* < 0.001).

### 2.2. Representative ^1^H-NMR Spectral Profiles

**Figure 1:**
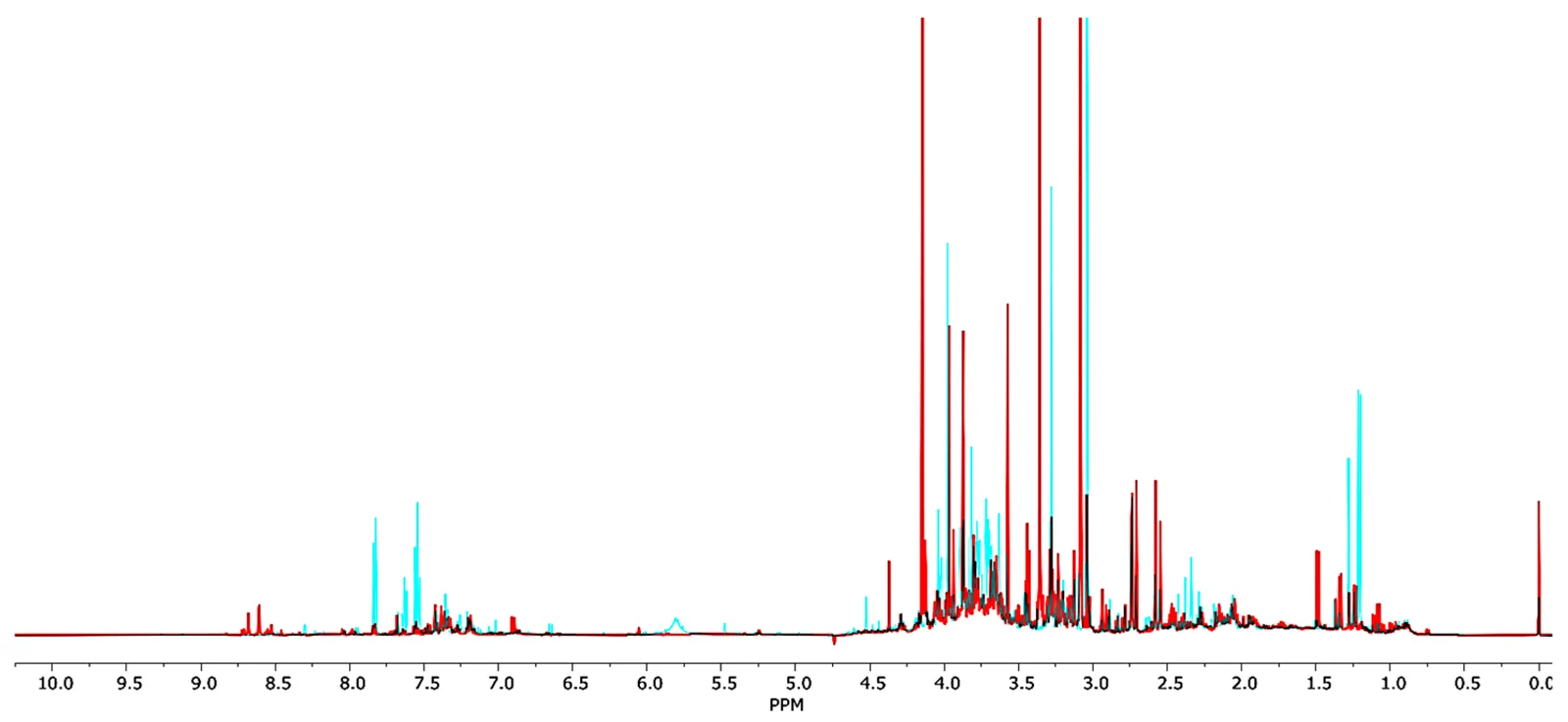
Representative overlaid urinary ^1^H-NMR spectra comparing participants from the high-exposure group (Chiang Mai; blue) and low-exposure group (Songkhla; red). The spectra provide an overview of the urinary metabolite profiles across the full chemical shift range.

Representative overlaid urinary ^1^H-NMR spectra from the high- and low-exposure groups are shown in Figure 1. Overall spectral patterns were broadly similar between groups, reflecting the complex composition of urinary metabolites. The observed resonance signals arise from multiple endogenous urinary metabolites that were putatively identified through spectral annotation during the metabolomics workflow. Visual inspection of the overlaid spectra demonstrated relative differences in signal intensities across several resonance regions between exposure groups. These qualitative observations are consistent with the statistically significant metabolomic differences identified through the subsequent multivariate and univariate analyses.

### 2.3. Multivariate Analysis of Urinary Metabolomic Profiles

**Figure 2:**
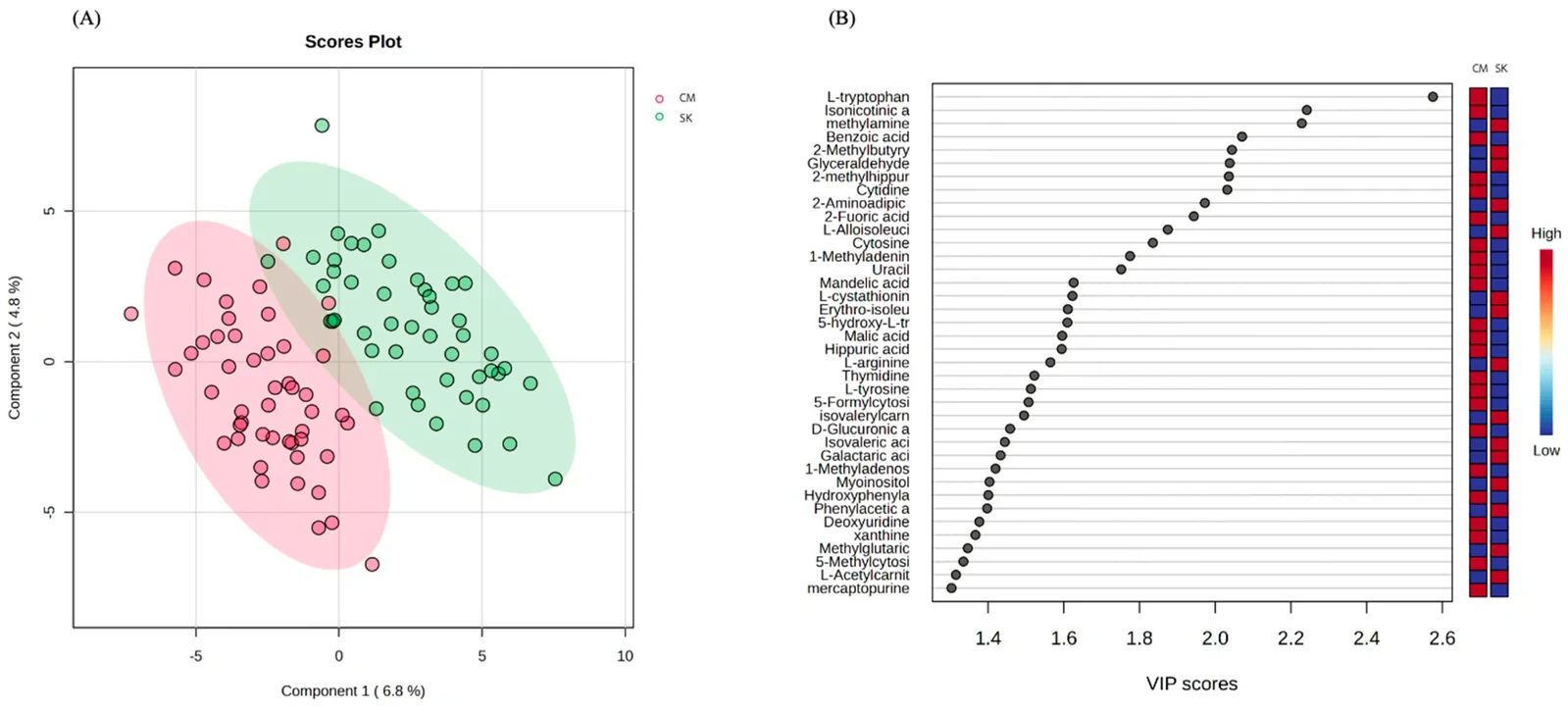
Partial least squares-discriminant analysis (PLS-DA) scores plot (**A**) and variable importance in projection (VIP) plot (**B**) comparing urinary metabolomic profiles between Chiang Mai (CM) and Songkhla (SK) groups.

Partial least squares-discriminant analysis (PLS-DA) was performed using MetaboAnalyst 6.0 to evaluate global differences in urinary metabolic profiles between participants in the high-exposure group (n = 51) and low-exposure group (n = 52). The PLS-DA score plot (Figure 2A) demonstrated a separation between the two groups, indicating distinct urinary metabolic signatures associated with contrasting ambient air pollution exposure environments.

Model performance was evaluated using cross-validation and permutation testing. The cross-validation results demonstrated good model fit (R^2^ = 0.873) and moderate predictive ability (Q^2^ = 0.447), with an overall classification accuracy of 87.4%. Permutation testing further supported model validity, as the observed classification statistic was significantly greater than those obtained from permuted models (*p* < 0.01; Supplementary Figure S1). These findings suggest moderate discriminatory performance of the multivariate model while reducing the likelihood of model overfitting. However, the predictive performance of the model should be interpreted with appropriate caution.

The first two latent components (Component 1 and Component 2) captured the main variation contributing to group separation and were used for visualization and interpretation. The corresponding variable importance in projection (VIP) plot (Figure 2B) identified metabolites with VIP scores ≥ 1.0 that contributed to the observed separation; the full list of these metabolites is provided in Supplementary Table S1. These metabolites included features that were both increased and decreased in the high-exposure group, indicating differences in urinary metabolite composition between exposure groups. 

### 2.4. Differential Metabolite Abundance Between Exposure Groups 

**Figure 3:**
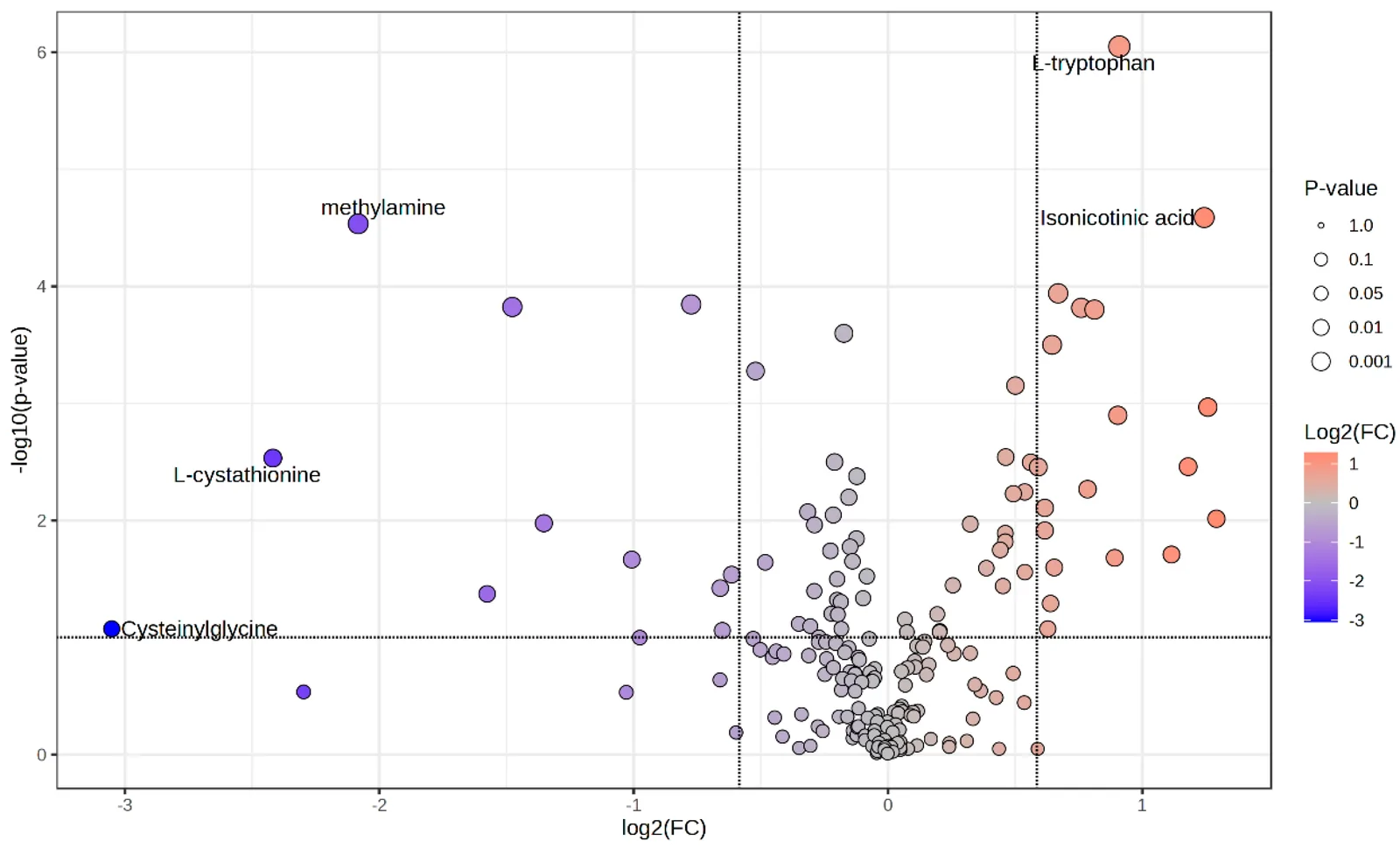
Volcano plot of differential urinary putative metabolites in the high-exposure group (Chiang Mai) relative to the low-exposure group (Songkhla). The horizontal dashed line indicates the significance threshold, and the vertical dashed lines indicate the fold-change thresholds.

Volcano plot analysis demonstrated multiple putative urinary metabolites differing between the high- and low-exposure groups (Figure 3). Metabolites presented in Table 2 were prioritized based on combined statistical significance (*p* < 0.05), contribution to group separation in the PLS-DA model (VIP ≥ 1.0), and biological relevance to exposure-associated metabolic pathways, while the complete metabolite list is provided in Supplementary Table S2. To account for multiple comparisons, false discovery rate (FDR) correction using the Benjamini–Hochberg method was additionally applied.

Positive log_2_ fold change values indicate higher relative abundance in the high-exposure group compared with the low-exposure group. L-tryptophan demonstrated the strongest statistical association, showing marked elevation in the high-exposure group (fold change = 1.878; log_2_FC = 0.909) together with the lowest FDR-adjusted *p*-value (FDR-adjusted *p* < 0.001). Uracil, malic acid, hippuric acid, L-cystathionine, and L-arginine also remained significant following FDR correction (FDR-adjusted *p* < 0.05). In contrast, xanthine and 5-hydroxyindoleacetic acid (5-HIAA) did not remain statistically significant after FDR correction (FDR-adjusted *p* = 0.071 and 0.096, respectively). Although VIP scores were used to identify candidate discriminatory metabolites, interpretation was further informed by univariate statistical testing, FDR-adjusted results, and biological relevance to exposure-associated metabolic pathways.

Among the prioritized metabolites, L-tryptophan, uracil, malic acid, hippuric acid, xanthine, and 5-HIAA showed higher relative abundance in the high-exposure group, whereas L-cystathionine and L-arginine showed lower relative abundance.

### 2.5. Receiver Operating Characteristic (ROC) Curve Analysis

**Figure 4:**
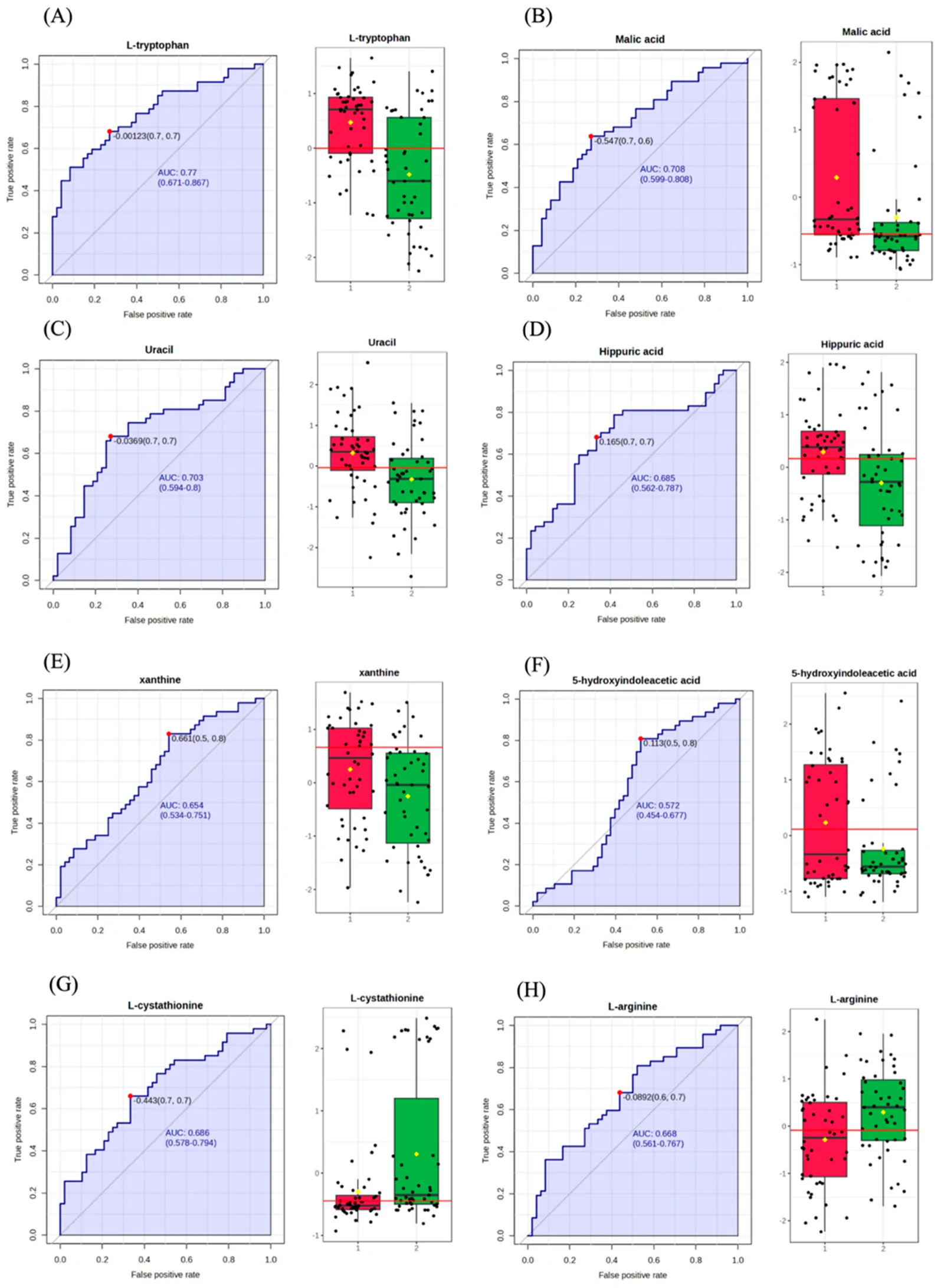
Receiver operating characteristic (ROC) curves and boxplots of selected urinary metabolites differentiating high-exposure (Chiang Mai) and low-exposure (Songkhla) PM_2.5_ groups. (**A**) L-tryptophan; (**B**) malic acid; (**C**) uracil; (**D**) hippuric acid; (**E**) xanthine; (**F**) 5-hydroxyindoleacetic acid; (**G**) L-cystathionine; and (**H**) L-arginine. Areas under the ROC curve (AUC) indicates classification performance between exposure groups. Boxplots display metabolite distributions in the high-exposure (Chiang Mai) and low-exposure (Songkhla) groups, shown in red and green, respectively.

Univariate ROC curve analysis was performed to evaluate the ability of individual urinary metabolites to distinguish between high- and low-exposure groups (Figure 4). Among metabolites showing higher relative abundance in the high-exposure group, L-tryptophan demonstrated the highest discriminatory performance (AUC = 0.770, 95% CI: 0.671–0.867), followed by malic acid (AUC = 0.708, 95% CI: 0.599–0.808), uracil (AUC = 0.703, 95% CI: 0.594–0.809), and hippuric acid (AUC = 0.685, 95% CI: 0.582–0.787). Xanthine and 5-hydroxyindoleacetic acid (5-HIAA) demonstrated lower discriminatory ability, with AUC values of 0.654 and 0.572, respectively.

Among metabolites showing lower relative abundance in the high-exposure group, L-cystathionine (AUC = 0.686, 95% CI: 0.578–0.794) and L-arginine (AUC = 0.668, 95% CI: 0.561–0.767) demonstrated moderate discriminatory ability.

Overall, most metabolites demonstrated limited-to-moderate discriminatory performance (AUC range approximately 0.57–0.77), indicating that individual metabolites alone have limited ability to distinguish exposure groups. These findings support the exploratory nature of the identified metabolic alterations and suggest that the detected metabolites should not be interpreted as standalone clinically predictive biomarkers.

### 2.6. Confounder-Adjusted Regression and Sensitivity Analyses

Multivariable linear regression analyses were performed to evaluate whether metabolite differences between exposure groups remained after adjustment for age, sex, body mass index (BMI), smoking status, and alcohol consumption (Table 3). After covariate adjustment and FDR correction, L-tryptophan (β = 9.62, 95% CI: 4.46–14.77), hippuric acid (β = 40.38, 95% CI: 9.34–71.42), xanthine (β = 9.51, 95% CI: 3.22–15.79), and 5-hydroxyindoleacetic acid (5-HIAA) (β = 220.26, 95% CI: 75.27–365.26) remained significantly associated with the high-exposure group. Although malic acid, uracil, L-arginine, and L-cystathionine showed estimated associations with exposure group after covariate adjustment, none remained statistically significant following FDR correction. Compared with the univariate analysis, these changes suggest that demographic and behavioral covariates contributed to variability in individual metabolite concentrations, thereby influencing the estimated associations with exposure group.

Sensitivity analyses restricted to non-drinkers demonstrated generally similar metabolite patterns between exposure groups (Supplementary Table S3). L-tryptophan, uracil, malic acid, and hippuric acid remained significant after FDR correction, whereas xanthine demonstrated a weak association. In contrast, 5-HIAA, L-arginine, and L-cystathionine were not significant in the sensitivity analyses. Overall, adjustment for alcohol consumption did not substantially alter the overall pattern of metabolite associations, although the statistical significance of several metabolites varied across analytical approaches.

Values are presented as regression coefficients (β) with 95% confidence intervals (CI) derived from multivariable linear regression models. Models were adjusted for age, sex, body mass index (BMI), smoking status, and alcohol consumption. Positive β coefficients indicate higher metabolite levels in the high-exposure group relative to the low-exposure group. FDR-adjusted *p*-values were calculated using the Benjamini–Hochberg false discovery rate correction method.

## 3. Discussion

### 3.1. Overview of Study and Main Findings

Applying untargeted ^1^H-NMR-based urinary metabolomics, we identified eight putative urinary metabolites that differed between adults residing in two Thai regions with contrasting ambient air pollution environments, defined by markedly different ambient PM_2.5_ concentrations. These metabolites were related to pathways involved in tryptophan metabolism, nucleotide metabolism, energy metabolism, gut–host co-metabolism, and amino acid metabolism, suggesting coordinated metabolic responses associated with differing environmental exposure conditions.

Overall, the findings indicate coordinated metabolomic alterations associated with contrasting ambient air pollution exposure environments. Because exposure classification was based on two geographically distinct regions, the observed metabolic differences should be interpreted as associations with differing environmental exposure conditions rather than effects attributable solely to PM_2.5_ mass concentration. The identified pathways converge on biological processes involved in redox regulation, providing biologically plausible hypotheses that warrant further investigation but do not establish underlying mechanisms.

Additional multivariable and sensitivity analyses demonstrated that some metabolites, particularly L-tryptophan and hippuric acid, showed more consistent associations across analytical approaches, whereas others were more sensitive to covariate adjustment, indicating that demographic and behavioral factors contributed to variability in individual metabolite concentrations. Given the cross-sectional design, these findings do not establish causality or temporal relationships and require confirmation in prospective studies.

### 3.2. Tryptophan-Serotonin Axis and Systemic Stress Responses

L-tryptophan was consistently elevated in the high-exposure group across multiple analytical approaches, while 5-hydroxyindoleacetic acid (5-HIAA), the principal metabolite of serotonin degradation [16], demonstrated less consistent statistical associations. Nevertheless, the concurrent directional increase in both metabolites may suggest potential alterations in the tryptophan–serotonin metabolic axis associated with contrasting ambient air pollution exposure environments.

Tryptophan metabolism is known to be sensitive to oxidative and inflammatory conditions, with both serotonergic and kynurenine pathways responding to changes in cellular redox status [15]. Therefore, the observed metabolic pattern may be consistent with altered serotonin turnover under environmental stress-related conditions. Peripheral serotonergic signaling has also been implicated in endothelial regulation, platelet activation, and vascular remodeling [11]. Accordingly, alterations in the tryptophan–serotonin axis may reflect broader systemic metabolic responses associated with contrasting ambient air pollution exposure environments. Because exposure classification was based on regional ambient PM_2.5_ concentrations rather than comprehensive pollutant characterization, the contribution of PM_2.5_ relative to other co-occurring environmental exposures cannot be distinguished. However, the present study did not directly assess inflammatory signaling, oxidative stress biomarkers, or serotonin metabolism, and these interpretations remain biologically plausible hypotheses that require further mechanistic investigation.

### 3.3. Nucleotide Metabolism and Cellular Stress Responses

Elevated levels of urinary xanthine and uracil in the high-exposure group suggest alterations in purine and pyrimidine metabolism [13]. Such changes may reflect increased nucleotide turnover or metabolic adaptation under conditions of environmental stress [12,13]. Xanthine is a product of purine degradation and is associated with enzymatic pathways capable of generating reactive oxygen species, whereas uracil reflects pyrimidine turnover and nucleic acid metabolism.

Previous studies have reported altered nucleotide metabolism in association with air pollution exposure [14,20]. In the present study, however, the statistical robustness of xanthine and uracil varied across analytical approaches. Uracil remained significant following FDR correction and sensitivity analysis but was attenuated after multivariable adjustment, whereas xanthine demonstrated significance primarily in covariate-adjusted models. These inconsistencies suggest that the observed associations may be influenced by interrelated demographic, behavioral, or metabolic factors and should therefore be interpreted cautiously.

Importantly, the present study did not directly measure oxidative DNA damage, nucleotide oxidation, or purine/pyrimidine turnover rates. Accordingly, these findings are more appropriately interpreted as exploratory metabolic patterns potentially related to redox-associated biological response rather than as a direct indicator of oxidative damage or nucleotide injury.

### 3.4. Hippuric Acid and Gut–Host Metabolic Interactions

Hippuric acid, a glycine conjugate of benzoic acid formed primarily in the liver, was elevated in the high-exposure group and remained consistently associated with exposure status across univariate, multivariable, and sensitivity analyses, suggesting that the observed association was relatively robust to adjustment for measured demographic and behavioral covariates. Hippuric acid reflects the combined influence of dietary intake, gut microbiota metabolism, and hepatic conjugation processes [29]. Elevated urinary hippuric acid may therefore reflect integrated metabolic responses associated with environmental exposure, including biotransformation of aromatic organic compounds derived from combustion-related air pollution. Previous studies have similarly linked PM_2.5_ exposure to metabolic perturbations involving gut–host interactions and oxidative stress-related pathways [17,30].

Nevertheless, hippuric acid is influenced by multiple external and endogenous factors, including diet, gut microbiota composition, and hepatic metabolism. Because these factors were not directly assessed in the present study, the specific determinants of increased hippuric acid levels cannot be fully established. Hippuric acid may, therefore, serve as a component of a broader metabolic response associated with environmental exposure rather than as a specific indicator of individual exposure pathways.

### 3.5. Energy Metabolism and Environmental Stress

Malic acid, an intermediate of the tricarboxylic acid (TCA) cycle, was elevated in the high-exposure group, suggesting alterations in cellular energy metabolism. The TCA cycle plays a central role in mitochondrial energy production and is sensitive to changes in cellular redox balance.

Previous studies have linked PM_2.5_ exposure to metabolic perturbations involving mitochondrial and energy-related pathways [31]. Therefore, the elevation of malic acid observed in this study may be consistent with broader metabolic responses associated with environmental stress exposure.

However, malic acid did not remain statistically significant after multivariable adjustment and FDR correction and was not consistently retained in sensitivity analyses. In addition, the present study did not directly assess mitochondrial function, oxidative stress biomarkers, or TCA cycle activity. Accordingly, these findings should be interpreted cautiously and considered exploratory rather than direct evidence of mitochondrial dysfunction or oxidative damage.

### 3.6. Alterations in L-Arginine and L-Cystathionine: Vascular and Redox Regulation

Reduced urinary L-arginine and L-cystathionine levels in the high-exposure group may suggest alterations in pathways related to nitric oxide metabolism and transsulfuration, respectively. These pathways are biologically relevant to vascular regulation and intracellular redox balance.

L-arginine serves as a substrate for nitric oxide synthesis and plays an important role in endothelial physiology [18]. Meanwhile, L-cystathionine participates in sulfur amino acid metabolism and glutathione-related pathways involved in antioxidant defense [19]. Previous studies have reported that oxidative and inflammatory conditions may influence these metabolic pathways [32].

However, the associations of L-arginine and L-cystathionine with exposure group were attenuated after multivariable adjustment and were not consistently retained in sensitivity analyses. In addition, the present study did not directly measure endothelial function, nitric oxide bioavailability, glutathione status, or oxidative stress biomarkers. Therefore, these findings should be interpreted cautiously as exploratory metabolic associations rather than evidence of endothelial dysfunction, impaired nitric oxide signaling, or antioxidant depletion.

Nevertheless, the observation of metabolites related to nitric oxide metabolism and transsulfuration in a relatively young and healthy population may support the hypothesis that subclinical metabolic alterations can occur in association with environmental exposure before overt cardiometabolic disease develops. Further longitudinal and mechanistic studies incorporating functional vascular and oxidative stress biomarkers are required to clarify the biological significance of these findings.

### 3.7. Integrated Metabolic Response to Contrasting Ambient Air Pollution Environments

Collectively, the identified metabolites suggest a coordinated metabolic alteration involving amino acid metabolism, nucleotide turnover, energy metabolism, and gut–host interactions. These pathways are interconnected and broadly related to cellular redox balance and systemic metabolic regulation. Because the exposure groups were defined by residence in two geographically distinct regions with contrasting ambient PM_2.5_ concentrations, the observed metabolic differences should be interpreted as associations with contrasting ambient air pollution environments rather than effects attributable solely to PM_2.5_ mass concentration. Other unmeasured pollutants and regional environmental factors may also have contributed to the observed metabolic patterns.

Importantly, the statistical robustness of the identified metabolites varied across analytical approaches. L-tryptophan and hippuric acid demonstrated the most consistent associations across univariate, multivariable, and sensitivity analyses, whereas several other metabolites showed weaker or less consistent associations after covariate adjustment or multiple-testing correction. These differences are likely attributable to the influence of demographic and behavioral covariates on individual metabolite concentrations. Adjustment for these covariates reduced unexplained inter-individual variability, strengthening the associations for some metabolites (e.g., xanthine and 5-HIAA), while attenuating others whose unadjusted associations were partly explained by these factors. These findings suggest that certain metabolic pathways may represent more robust exposure-associated signals than others.

The moderate discriminatory performance observed in ROC analyses further suggests that metabolic responses associated with contrasting ambient air pollution exposure environments are multifactorial and are better characterized at the pathway level than by individual metabolites alone. Accordingly, the present findings should be interpreted as exploratory metabolic patterns associated with exposure status rather than definitive biomarkers of PM_2.5_ exposure or specific biological mechanisms. Future longitudinal studies incorporating comprehensive exposure assessment, characterization of ambient pollutants, and targeted metabolite quantification will be essential to clarify the biological relevance of these metabolic alterations and their relationship to ambient air pollution exposure.

### 3.8. Study Limitations and Public Health Implications

Several limitations should be considered when interpreting these findings. Metabolite annotations were based on one-dimensional ^1^H-NMR profiling and should be considered putative, particularly for low-abundance metabolites and structurally overlapping signals [33]. Because HMDB matching provides candidate annotations based on spectral similarity, some assignments may represent biologically implausible or alternative metabolites rather than the true compounds present in the samples. Accordingly, metabolite identities should be interpreted cautiously and require confirmation using complementary analytical approaches such as LC–MS/MS. In addition, formal analytical validation parameters, including metabolite-specific reproducibility metrics (e.g., QC-based RSD%), PCA-based assessment of QC clustering, LOD/LOQ determination, recovery, precision testing, and absolute quantification using isotope-labeled standards, were not comprehensively evaluated.

The cross-sectional design precludes causal inference, and regional residence was used as a proxy for PM_2.5_ exposure rather than individual-level exposure assessment. Because exposure classification was based on two geographically distinct regions, some of the observed metabolic differences may have been influenced by unmeasured regional factors, including socioeconomic characteristics, dietary habits, lifestyle characteristics, climate, cultural practices, or other environmental exposures. Therefore, the identified metabolic alterations should be interpreted as associations with contrasting ambient air pollution exposure environments rather than effects attributable exclusively to PM_2.5_ mass concentration. In addition, detailed characterization of PM_2.5_ chemical composition was not available; therefore, potential contributions from differences in particle constituents between regions could not be evaluated.

In addition, although multivariable analyses adjusted for age, sex, BMI, smoking status, and alcohol consumption, residual confounding from unmeasured factors cannot be excluded. Although all participants underwent standardized overnight fasting and morning urine collection to minimize pre-analytical variability, urine dilution was not adjusted using creatinine, specific gravity, or osmolality measurements. Spectral data were normalized using sample median normalization to reduce systematic variation in overall signal intensity among samples; however, this approach cannot fully account for inter-individual differences in urine dilution or hydration status. Consequently, hydration-related variability may have contributed to inter-individual differences in urinary metabolite concentrations and affected relative metabolite quantification and spectral interpretation. Therefore, the present findings should be regarded as exploratory. Future studies should incorporate established urine dilution measures to enable complementary dilution-corrected analyses.

The study also lacked direct biomarkers of oxidative stress, endothelial function, or mitochondrial dysfunction. Therefore, mechanistic interpretations linking metabolite alterations to oxidative stress, vascular dysfunction, or redox imbalance remain inferential and should be considered biologically plausible hypotheses rather than demonstrated conclusions. Furthermore, several metabolites showed variable statistical robustness across univariate, multivariable, and sensitivity analyses, indicating that some associations should be interpreted cautiously. In addition, ROC analyses were performed using the same dataset from which the metabolites were identified and were not externally validated. Therefore, the reported AUC values should be considered exploratory and should not be interpreted as evidence of validated biomarker performance. Despite these limitations, the present study identified coordinated metabolic alterations associated with contrasting ambient air pollution exposure environments in apparently healthy young adults, supporting the potential utility of urinary metabolomics for characterizing early biological responses to environmental exposure.

## 4. Materials and Methods

### 4.1. Study Design, Population, and PM_2.5_ Exposure Classification

A cross-sectional comparative study design was employed to evaluate the associations between ambient PM_2.5_ exposure conditions and urinary metabolite profiles. Participants were recruited from two regions of Thailand with contrasting ambient PM_2.5_ levels: Chiang Mai province in northern Thailand (high exposure) and Songkhla province in southern Thailand (low exposure). A total of 103 adults aged 20–40 years were enrolled, including 51 participants from Chiang Mai and 52 from Songkhla. Eligible participants were required to have continuously resided and worked within their respective study areas for at least three years prior to recruitment to support stable regional exposure classification.

Ambient PM_2.5_ concentration data were obtained from the publicly available database of the Thailand PCD monitoring network (https://www.pcd.go.th). Exposure classification was based on data from the Yupparaj School monitoring station (36T) in Chiang Mai and the Hat Yai monitoring station (44T) in Songkhla, which are located within the participant recruitment areas. Daily 24 h average PM_2.5_ concentrations recorded during the urine collection period (February–April 2025) were used to characterize regional ambient air pollution conditions. During this period, the highest recorded 24 h average PM_2.5_ concentration in Chiang Mai was approximately 60 µg/m^3^, exceeding both the World Health Organization (WHO) guideline (15 µg/m^3^) and the Thai national standard (37.5 µg/m^3^), whereas the corresponding value in Songkhla was approximately 20 µg/m^3^. These regional monitoring data were used to classify Chiang Mai as the high-exposure region and Songkhla as the low-exposure region. 

Participants were excluded if they had acute infections within 30 days prior to recruitment, were pregnant or breastfeeding, or had known chronic medical conditions or regular medication use that could influence metabolic profiling. Ethical approval was obtained from the Research Institute for Health Sciences Ethics Committee, Chiang Mai University (No 38/2024, Project No. 16/67), and written informed consent was obtained from all participants prior to enrollment.

### 4.2. Chemical Reagents

All chemical reagents used were of analytical grade. Deuterium oxide (D_2_O) and trimethylsilyl propanoic acid (TSP) were obtained from Sigma-Aldrich (St. Louis, MO, USA), while acetonitrile was purchased from Merck (Darmstadt, Germany).

### 4.3. Preparation of Urine Samples

Following a 12 h overnight fast, mid-stream morning urine samples were collected from all participants using a standardized collection protocol and stored at −20 °C until analysis. Prior to analysis, samples were thawed, vortexed, and centrifuged at 15,000× *g* for 3 min to remove particulates. A 0.6 mL aliquot of supernatant was mixed with 0.6 mL of D_2_O containing 0.1 mM TSP, which served as an internal standard for chemical shift referencing and relative quantification. ^1^H-NMR spectra were obtained on a 500 MHz spectrometer with water suppression. Urinary creatinine, specific gravity, and osmolality were not measured; therefore, urine dilution was not corrected during data preprocessing.

### 4.4. ^1^H-NMR Spectroscopy

^1^H-NMR spectra were acquired using a Bruker AVANCE 500 MHz spectrometer (Bruker, Bremen, Germany) equipped with a Carr–Purcell–Meiboom–Gill (CPMG; RD-90°, (τ-180°) n-acquire) pulse sequence. Water signal suppression was achieved by presaturation, and all spectra were recorded at 27 °C. The acquisition parameters were as follows: spectral width of 8278.146 Hz, free induction decay (FID) resolution of 0.126 Hz, dwell time of 60.40 µs, relaxation delay of 1 s, acquisition time of 3.95 s, and 16 scans. Spectral processing, including phase and baseline correction, was performed using TopSpin software (version 4.0.7). Spectra were examined over the chemical shift range of 0–12 ppm. Metabolite signals were identified by comparing chemical shift patterns with entries in established human metabolite databases. 

Trimethylsilyl propanoic acid (TSP) was used as the internal standard for chemical shift calibration at 0.00 ppm and for relative metabolite quantification. TSP was selected because it produces a sharp and well-resolved resonance signal and is commonly used in ^1^H-NMR-based metabolomic analyses.

### 4.5. Spectral Interpretation and Metabolite Identification

Metabolites were identified by matching the chemical shifts observed in the ^1^H-NMR spectra with reference values in the Human Metabolome Database (HMDB). Spectral analysis was conducted using Bruker TopSpin software (version 4.0.7), including peak picking, signal integration, and J-coupling analysis. Assignments were made based on combined agreement of chemical shift, spin-spin coupling patterns, and spectral consistency across samples. Putative metabolite annotation was considered acceptable when the observed chemical shifts agreed with HMDB references within ±0.01 ppm. Metabolite assignments were considered putative and were not confirmed using complementary 2D NMR or LC–MS/MS analyses.

### 4.6. Quality Control

Quality control (QC) samples were prepared by pooling 5 µL aliquots from each urine sample to generate a representative composite sample. QC samples were analyzed at the beginning of the analytical sequence and periodically (every 20–23 samples) to monitor instrument stability and analytical reproducibility. All samples were analyzed in randomized order to minimize systematic bias. Blank and solvent controls were included to detect background signals or contamination. Key NMR quality metrics, including signal-to-noise ratio, line width, and chemical shift consistency, were evaluated using dedicated NMR processing software (MestReNova and Bruker TopSpin) to ensure analytical robustness and data reliability. Analytical reproducibility was evaluated by calculating the relative standard deviation (RSD) of metabolite peak intensities across repeated pooled QC measurements. The analytical performance metrics are summarized in Supplementary Table S4.

### 4.7. Calculation of Relative Concentration

^1^H-NMR spectra were processed and visualized using MestReNova software (version 14.1.2-25024, MestreLab Research, Santiago de Compostela, Spain). Metabolite signal intensities were calculated relative to the internal standard (TSP). following previously described NMR-based approaches by Jaikang, C., et al., [8,20]. Relative metabolite levels were calculated based on signal intensity using the following equation:

(1) $$I_{A}=\frac{H_{A}\times C_{A}}{H_{B}\times C_{B}}\times I_{B}$$

In this equation, I_A_ denotes the signal intensity of the metabolite of interest, I_B_ represents the signal intensity of TSP, H_A_ and H_B_ denote the number of equivalent hydrogen atoms contributing to the metabolite and TSP signals, respectively, C_A_ represents the calculated relative metabolite level, and C_B_ represents the concentration of TSP (µM). The calculated values represent relative metabolite levels rather than absolute metabolite concentrations.

### 4.8. Metabolomics Data Analysis

Metabolomics data were analyzed using MetaboAnalyst version 6.0 (https://www.metaboanalyst.ca) accessed on 19 December 2025. Prior to analysis, non-negative values and missing values were verified. No missing values were present in the final dataset used for analysis. The data were median-normalized to reduce systematic variation in overall signal intensity among samples, followed by log_10_ transformation and auto-scaling to improve comparability across metabolites.

Partial least squares-discriminant analysis (PLS-DA) was conducted to visualize and assess global metabolic differences between high-exposure (Chiang Mai) and low-exposure (Songkhla) groups. Model performance was evaluated using 10-fold cross-validation with 100 repetitions and permutation testing (100 permutations). The first two latent components were used for visualization and interpretation. Variable importance in projection (VIP) scores were computed to identify metabolites contributing most strongly to group separation, with a threshold of VIP ≥ 1.0 used to identify candidate discriminatory metabolites. Candidate metabolites were subsequently evaluated using univariate statistical testing with Benjamini–Hochberg false discovery rate (FDR) correction and multivariable regression analyses. Univariate receiver operating characteristic (ROC) curve analysis was performed to evaluate the ability of individual metabolites to discriminate between exposure groups. The area under the ROC curve (AUC) was used as a measure of classification performance.

### 4.9. Statistical Analysis

Participant characteristics were summarized using descriptive statistics. Continuous variables were presented as median (interquartile range, IQR), and categorical variables as counts (percentages). Differences between high- and low-exposure groups were evaluated using the Mann–Whitney U test for continuous variables and Fisher’s exact test for categorical variables. All statistical tests were two-sided, and *p*-values < 0.05 were considered statistically significant. 

Differential metabolite abundance between exposure groups was evaluated using fold change analysis and univariate statistical testing. Normality of metabolite distributions was assessed using the Shapiro–Wilk test. Because several metabolites did not satisfy the assumption of normality, the non-parametric Mann–Whitney U test was used for univariate comparisons between exposure groups. To account for multiple comparisons, *p*-values were adjusted using the Benjamini–Hochberg false discovery rate (FDR) procedure. Statistical significance was defined as a two-sided *p*-value < 0.05, and FDR-adjusted *p*-values < 0.05 were considered statistically significant. 

Multivariable linear regression analyses were additionally performed to evaluate associations between exposure group and individual metabolite levels. In these models, metabolite levels were treated as the dependent variables, whereas exposure group (high- versus low-exposure region) was included as the primary independent variable. Models were adjusted for age, sex, body mass index (BMI), smoking status, and alcohol consumption. Sensitivity analyses restricted to non-drinkers were conducted to further assess the potential influence of alcohol consumption on metabolite associations. 

Univariate receiver operating characteristic (ROC) curve analysis was performed to evaluate the discriminatory performance of individual metabolites between exposure groups. Statistical analyses, including Mann–Whitney U testing, false discovery rate adjustment, and multivariable linear regression, were conducted using R software (version 4.5.0) with the tidyverse, janitor, and broom packages. Metabolomics data preprocessing and multivariate analyses were performed using MetaboAnalyst (version 6.0).

## 5. Conclusions

This study identified coordinated urinary metabolomic alterations associated with residence in two regions characterized by contrasting ambient air pollution exposure environments, rather than with PM_2.5_ mass concentration alone, in a population of apparently healthy Thai adults. The observed differences in urinary metabolite profiles expand current knowledge of metabolic alterations associated with contrasting environmental exposure conditions and demonstrate the potential of urinary metabolomics for investigating early biological responses to ambient air pollution.

These findings suggest coordinated metabolic responses associated with contrasting ambient air pollution exposure environments. However, given the cross-sectional design, the results represent associations rather than causal relationships. In addition, metabolite annotations were putative, several metabolites showed variable statistical robustness across analytical approaches, and the proposed biological mechanisms remain hypotheses requiring further validation.

Overall, this study provides exploratory evidence of exposure-associated metabolic alterations in a young and apparently healthy population and supports the potential utility of urinary metabolomics for characterizing early biological responses to environmental exposure. Because exposure classification was based on residence in two geographically distinct regions, some of the observed metabolic differences may have been influenced by unmeasured regional factors, including socioeconomic characteristics, dietary habits, lifestyle factors, climate, cultural practices, and workplace-specific or indoor air pollution exposures. Although participants’ occupations were recorded, detailed information on workplace-specific exposure conditions and indoor air quality was not available. Therefore, residual confounding cannot be excluded. Future longitudinal studies incorporating individual-level exposure assessment, comprehensive characterization of ambient pollutants, targeted metabolite validation, and functional biomarkers, and detailed assessment of dietary and lifestyle factors are warranted to clarify the biological and clinical relevance of these metabolic alterations.

## Figures and Tables

**Table 1:** Demographic data of participants in Chiang Mai and Songkhla.

Characteristic	Chiang Mai (CM)	Songkhla (SK)	*p*-Value
Age (years)	30.0 (25.00–35.00)	29.0 (25.75–35.25)	0.954
Sex, n (%)			
Male	21 (41.2%)	27 (51.9%)	0.238
Alcohol consumption, n (%)	41 (80.4%)	23 (44.2%)	0.0002 **
Smoking, n (%)	8 (15.7%)	4 (7.7%)	0.235
BMI (kg/m^2^)	24.6 (22.3–26.5)	24.1 (21.9–26.7)	0.465
Blood Pressure (mmHg)			
Systolic Blood Pressure	119.0 (110.0–127.0)	114.5 (105.0–123.3)	0.076
Diastolic Blood Pressure	78.0 (75.0–85.8)	79.0 (71.0–83.0)	0.559
Heart rate (bpm)	76.0 (67.5–86.0)	81.0 (76.5–88.0)	0.062
Occupation			0.121
Unemployed	1 (2.0)	6 (11.5)	
Daily-wage worker	1 (2.0)	1 (1.9)	
Private sector employees	15 (29.4)	6 (11.5)	
Government employees	25 (49.0)	28 (53.8)	
Self-employed	2 (3.9)	1 (1.9)	
**Other**	7 (13.7)	10 (19.2)	

Data are presented as median (interquartile range, IQR) or n (%), as appropriate. Continuous variables were compared using the Mann–Whitney U test, and categorical variables were compared using Fisher’s exact test. A two-side *p*-values < 0.05 were considered statistically significant. ** *p* < 0.01.

**Table 2:** Differential urinary putative metabolites between high- and low-exposure groups.

Metabolite	VIP (Comp. 1)	VIP (Comp. 2)	Fold Change (High/Low)	log_2_(FC)	*p*-Value	FDR-Adjusted *p*-Value
L-Tryptophan	2.575	2.265	1.878	0.909	<0.001	<0.001
Uracil	1.752	1.581	1.870	0.903	0.001	0.017
L-Cystathionine	1.623	1.467	0.187	−2.418	0.003	0.033
Malic acid	1.596	1.390	2.266	1.180	0.003	0.033
Hippuric acid	1.594	1.495	1.506	0.591	0.003	0.033
L-Arginine	1.565	1.464	0.918	−0.123	0.004	0.038
Xanthine	1.366	1.214	1.377	0.461	0.013	0.071
5-Hydroxyindoleacetic acid	1.272	1.591	1.854	0.891	0.021	0.096

VIP: variable importance in projection derived from the partial least squares-discriminant analysis (PLS-DA) model. Fold change values > 1 indicate higher relative abundance in the high-exposure group (Chiang Mai), whereas values < 1 indicate lower relative abundance. Positive log_2_(FC) values indicate higher abundance in the high-exposure group. FDR-adjusted *p*-values were calculated using the Benjamini–Hochberg false discovery rate correction method.

**Table 3:** Multivariable linear regression analyses of urinary metabolites associated with high- and low-PM_2.5_ exposure groups after adjustment for demographic and behavioral covariates.

Metabolite	β Coefficient	95% CI	*p*-Value	FDR-Adjusted *p*-Value
L-Tryptophan	9.62	4.46–14.77	<0.001	0.003
Uracil	141.48	−78.96–361.92	0.206	0.235
L-Cystathionine	−1658.96	−3617.74–299.82	0.096	0.128
Malic acid	169.95	10.16–329.75	0.037	0.060
Hippuric acid	40.38	9.34–71.42	0.011	0.023
L-Arginine	2.27	−4.69–9.23	0.518	0.518
Xanthine	9.51	3.22–15.79	0.003	0.009
5-Hydroxyindoleacetic acid	220.26	75.27–365.26	0.003	0.009

## Data Availability

The original contributions presented in the study are included in this article and Supplementary Materials. Anonymized metabolite-intensity data, relevant participant covariates, and analysis details are available from the corresponding author upon reasonable request, subject to ethical approval and participant consent restrictions. PM_2.5_ exposure data are publicly available from the Thailand Pollution Control Department (PCD) monitoring network (https://www.pcd.go.th).

## Supplementary Materials

The following supporting information can be downloaded at: https://www.mdpi.com/article/10.3390/ijms27167193/s1.

## Abbreviations

The following abbreviations are used in this manuscript:

5-HIAA	5-Hydroxyindoleacetic acid
AhR	Aryl hydrocarbon receptor
AUC	Area under the curve
CSE	Cystathionine gamma-lyase
eNOS	Endothelial nitric oxide synthase
FDR	False discovery rate
GSH	Glutathione
^1^H-NMR	Proton nuclear magnetic resonance
NO	Nitric oxide
PCD	Pollution Control Department
PLS-DA	Partial least squares-discriminant analysis
PM_2.5_	Fine particulate matter (≤2.5 µm)
ROC	Receiver operating characteristic
ROS	Reactive oxygen species
TCA	Tricarboxylic acid
TSP	Trimethylsilyl propanoic acid
VIP	Variable importance in projection

## References

[1] Wang W., Wang H., Zhao R. (2025). The burden of stroke attributable to ambient particulate matter pollution in China: Findings from the Global Burden of Disease Study 2021. Int. J. Environ. Health Res..

[2] Jalali S., Karbakhsh M., Momeni M., Taheri M., Amini S., Mansourian M., Sarrafzadegan N. (2021). Long-term exposure to PM_2.5_ and cardiovascular disease incidence and mortality in an Eastern Mediterranean country: Findings based on a 15-year cohort study. Environ. Health.

[3] Thangavel P., Park D., Lee Y.-C. (2022). Recent insights into particulate matter (PM_2.5_)-mediated toxicity in humans: An overview. Int. J. Environ. Res. Public Health.

[4] Kumar V., S H., Huligowda L.K.D., Umesh M., Chakraborty P., Thazeem B., Singh A.P. (2025). Environmental Pollutants as Emerging Concerns for Cardiac Diseases: A Review on Their Impacts on Cardiac Health. Biomedicines.

[5] Suriyawong P., Chuetor S., Samae H., Piriyakarnsakul S., Amin M., Furuuchi M., Hata M., Inerb M., Phairuang W. (2023). Airborne particulate matter from biomass burning in Thailand: Recent issues, challenges, and options. Heliyon.

[6] Abdel-Shafy H.I., Mansour M.S.M. (2016). A review on polycyclic aromatic hydrocarbons: Source, environmental impact, effect on human health and remediation. Egypt. J. Pet..

[7] Aguilera R., Corringham T., Gershunov A., Leibel S., Benmarhnia T. (2021). Fine Particles in Wildfire Smoke and Pediatric Respiratory Health in California. Pediatrics.

[8] Jaikang C., Konguthaithip G., Amornlertwatana Y., Autsavapromporn N., Rattanachitthawat S., Monum T. (2024). Alterations in the blood kynurenine pathway following long-term PM2.5 and PM10 exposure: A cross-sectional study. Biomedicines.

[9] Buya S., Gokon H., Huynh V.-N., Dam H.-C., Usanavasin S., Karnjana J., Taneepanichskul N. (2026). Spatiotemporal association between monthly PM2.5 levels and cardiorespiratory mortality in Thailand (2015–2019). Int. J. Environ. Health Res..

[10] Lobato S., Salomón-Soto V.M., Espinosa-Méndez C.M., Herrera-Moreno M.N., García-Solano B., Pérez-González E., Comba-Marcó-del-Pont F., Montesano-Villamil M., Mora-Ramírez M.A., Mancilla-Simbro C. (2024). Molecular Pathways Linking High-Fat Diet and PM_2.5_ Exposure to Metabolically Abnormal Obesity: A Systematic Review and Meta-Analysis. Biomolecules.

[11] Ayme-Dietrich E., Aubertin-Kirch G., Maroteaux L., Monassier L. (2017). Cardiovascular remodeling and the peripheral serotonergic system. Arch. Cardiovasc. Dis..

[12] Borska L., Andrys C., Krejsek J., Palicka V., Chmelarova M., Hamakova K., Kremlacek J., Fiala Z. (2014). Oxidative damage to nucleic acids and benzo(a)pyrene-7,8-diol-9,10-epoxide-DNA adducts and chromosomal aberration in children with psoriasis repeatedly exposed to crude coal tar ointment and UV radiation. Oxidative Med. Cell Longev..

[13] Furuhashi M. (2020). New insights into purine metabolism in metabolic diseases: Role of xanthine oxidoreductase activity. Am. J. Physiol. Endocrinol. Metab..

[14] Li R., Zhao L., Zhang L., Chen M., Shi J., Dong C., Cai Z. (2017). Effects of ambient PM_2.5_ and 9-nitroanthracene on DNA damage and repair, oxidative stress and metabolic enzymes in the lungs of rats. Toxicol. Res..

[15] Salminen A. (2023). Activation of aryl hydrocarbon receptor (AhR) in Alzheimer’s disease: Role of tryptophan metabolites generated by gut host-microbiota. J. Mol. Med..

[16] Takahashi T., Yano M., Minami J., Haraguchi T., Koga N., Higashi K., Kobori S. (2002). Sarpogrelate hydrochloride, a serotonin2A receptor antagonist, reduces albuminuria in diabetic patients with early-stage diabetic nephropathy. Diabetes Res. Clin. Pract..

[17] Gonzalez M., Clayton S., Wauson E., Christian D., Tran Q.K. (2025). Promotion of nitric oxide production: Mechanisms, strategies, and possibilities. Front. Physiol..

[18] Lorin J., Zeller M., Guilland J.C., Cottin Y., Vergely C., Rochette L. (2014). Arginine and nitric oxide synthase: Regulatory mechanisms and cardiovascular aspects. Mol. Nutr. Food Res..

[19] Karadima E., Chavakis T., Alexaki V.I. (2025). Arginine metabolism in myeloid cells in health and disease. Semin. Immunopathol..

[20] Jaikang C., Konguthaithip G., Amornlertwatana Y., Autsavapromporn N., Rattanachitthawat S., Liampongsabuddhi N., Monum T. (2025). Metabolic Disruptions and Non-Communicable Disease Risks Associated with Long-Term Particulate Matter Exposure in Northern Thailand: An NMR-Based Metabolomics Study. Biomedicines.

[21] Zhu X., Tang X., Ng C., Li L., Lai Y., Miller G.W., Jiang C., Barchowsky A., Buchanich J., Gao P. (2025). Biomonitoring xenobiotics in human biospecimens: Challenges, advances, and the future of exposome characterization. Rev. Environ. Contam. Toxicol..

[22] Picone G. (2025). The Application of NMR-Based Metabolomics in the Field of Nutritional Studies. Encyclopedia.

[23] Deng P., Li X., Petriello M.C., Wang C., Morris A.J., Hennig B. (2019). Application of metabolomics to characterize environmental pollutant toxicity and disease risks. Rev. Environ. Health.

[24] Fang L., Kim I.S., Martin B.R., Sherlock L., Min S., Kim Y.K., Mok K.H. (2026). Metabolomics and Human Health: Progress, Insights, Challenges and the Concept of a Healthy Metabolome. Biomolecules.

[25] Carmen B. (2022). Metabolomics in environmental toxicology: Applications and challenges. Trends Environ. Anal. Chem..

[26] Li Y., Bansal S., Singh B., Jayatilake M.M., Klotzbier W., Boerma M., Lee M.-H., Hack J., Iwamoto K.S., Schaue D. (2024). Distinct Urinary Metabolite Signatures Mirror In Vivo Oxidative Stress-Related Radiation Responses in Mice. Antioxidants.

[27] Mukli P., Wu D.H., Csipo T., Owens C.D., Lipecz A., Racz F.S., Zouein F.A., Tabak A., Csiszar A., Ungvari Z. (2022). Urinary biomarkers of oxidative stress in aging: Implications for prediction of accelerated biological age in prospective cohort studies. Oxidative Med. Cell. Longev..

[28] Ni Q., Xia L., Huang Y., Yuan X., Gu W., Chen Y., Wang Y., Nian M., Wu S., Cai H. (2025). Gut microbiota dysbiosis orchestrates vitiligo-related oxidative stress through the metabolite hippuric acid. Microbiome.

[29] Ticinesi A., Guerra A., Nouvenne A., Meschi T., Maggi S. (2023). Disentangling the Complexity of Nutrition, Frailty and Gut Microbial Pathways during Aging: A Focus on Hippuric Acid. Nutrients.

[30] Maeda Y., Matsunaga M., Hosaka Y., Michii S., Hattori N., Ishikawa K., Ota A. (2026). Climatic effects on urinary hippuric acid concentrations: A 3-year occupational health study in a manufacturing workforce. J. Occup. Health.

[31] Wang D., Liu C., Ko Y.A., Chang H.H., Jin Z., Tan Y., Tran V., Jones D.P., Quyyumi A.A., Liang D. (2025). Effects of Exposure to Ambient Air Pollution on Human Metabolome in Patients with Coronary Artery Disease. Environ. Sci. Technol..

[32] Wang J., Jia G., Li H., Yan S., Qian J., Guo X., Li G., Qi H., Zhu Z., Wu Y. (2021). H_2_O_2_-Mediated Oxidative Stress Enhances Cystathionine γ-Lyase-Derived H_2_S Synthesis via a Sulfenic Acid Intermediate. Antioxidants.

[33] Andra S.S., Austin C., Patel D., Dolios G., Awawda M., Arora M. (2017). Trends in the application of high-resolution mass spectrometry for human biomonitoring: An analytical primer to studying the environmental chemical space of the human exposome. Environ. Int..

