# Supplementary material for: Urinary Metabolomic Alterations Associated with Contrasting Ambient Air Pollution Exposure in Thai Adults: An Untargeted 1H-NMR Study
Source: Int J Mol Sci. 2026 Aug 12;27(16):7193. doi: 10.3390/ijms27167193 (PMC13513067; doi:10.3390/ijms27167193)
Supplement: Supplementary file 1 [file ijms-27-07193-s001.zip › ijms-4437395-supplementary.pdf]

Urinary Metabolomic Alterations Associated with Contrasting Ambient Air Pollution Exposure in Thai Adults: An Untargeted ^{1}H-NMR Study

Blecious Zinan'dala [1,2], Anupon Iadnut [3,4], Chikondi Maluwa [1,2], Puriwat Fakfum [1], Churdsak Jaikang [5,6], Giatgong Konguthaithip [5,6], Kanokwan Kulprachakarn [1,3], Wason Parklak [1,3] and Hataichanok Chuljerm [1,3,*]

1 School of Health Sciences Research, Research Institute for Health Sciences, Chiang Mai University, Chiang Mai 50200, Thailand; dysonblecious_zina@cmu.ac.th (B.Z.); chikondi_maluwa@cmu.ac.th (C.M.); puriwat_f@cmu.ac.th (P.F.); kanokwan.kul@cmu.ac.th (K.K.); wason.p@cmu.ac.th (W.P.)
2 Faculty of clinical Sciences, Malawi College of Health Sciences, Lilongwe 3, Lilongwe P.O. Box 30368, Malawi
3 Research Institute for Health Sciences, Chiang Mai University, Chiang Mai 50200, Thailand; anupon_iad@nation.ac.th
4 Faculty of Medicine, Nation University, Chiang Mai 50210, Thailand
5 Department of Forensic Medicine, Faculty of Medicine, Chiang Mai University, Chiang Mai 50200, Thailand; churdsak.j@cmu.ac.th (C.J.); kongkiat.shang@gmail.com (G.K.)
6 Metabolomic Research Group for Forensic Medicine and Toxicology, Department of Forensic Medicine, Faculty of Medicine, Chiang Mai University, Chiang Mai 50200, Thailand
* Correspondence: hataichanok.ch@cmu.ac.th; Tel.: +66-5393-6148

Supplementary Table S1 Metabolites with VIP ≥ 1.0 identified by PLS-DA

**Metabolite**	**VIP score**	**Direction (high vs low exposure)**
L-tryptophan	2.61	Increased
Methylamine	2.26	Decreased
2-Methylbutyrylglycine	2.22	Decreased
Benzoic acid	2.14	Increased
2-Methylhippuric acid	2.03	Increased
Cytidine	1.99	Increased
2-Aminoadipic acid	1.94	Increased
L-Alloisoleucine	1.91	Decreased
2-Fluoric acid	1.88	Increased
1-Methyladenine	1.86	Increased
Cytosine	1.82	Increased
Uracil	1.71	Increased
L-Cystathionine	1.69	Decreased
Mandelic acid	1.66	Increased
L-Tyrosine	1.64	Increased
Hippuric acid	1.62	Increased
5-Hydroxy-L-tryptophan	1.60	Increased
1-Methyladenosine	1.59	Increased
Erythro-isoleucine	1.58	Decreased
L-Arginine	1.56	Decreased
Thymidine	1.54	Decreased
Malic acid	1.53	Increased

Phenylacetic acid	1.52	Decreased
Galactaric acid	1.49	Decreased
5-Formylcytosine	1.47	Increased
Isovaleric acid	1.46	Decreased
Isovalerylcarnitine	1.45	Decreased
D-Glucuronic acid	1.43	Decreased
Deoxyuridine	1.41	Increased
L-Acetylcarnitine	1.40	Decreased
Xanthine	1.38	Increased
Syringic acid	1.37	Increased
Myo-inositol	1.35	Decreased
5-Methylcytosine	1.34	Increased
Methylglutaric acid	1.33	Decreased
5-Hydroxyindoleacetic acid	1.32	Increased

Supplementary Table S2 Differentially abundant urinary metabolites identified by volcano plot analysis between high- and low-exposure groups

Metabolite	FC	$\log_2$(FC)	p value	FDR-adjusted *p*-value	$-\log_{10}$(p)
L-tryptophan	1.878	0.909	8.94E-07	<0.001	6.049
methylamine	0.236	-2.083	2.92E-05	0.003	4.535
Benzoic acid	1.590	0.669	1.15 E-04	0.005	3.940
2-Methylbutyrylglycine	0.585	-0.774	1.43 E-04	0.005	3.846
2-methylhippuric acid	1.693	0.759	1.53 E-04	0.005	3.817
Cytidine	1.755	0.812	1.58 E-04	0.005	3.802
2-Fuoric acid	1.564	0.645	3.16 E-04	0.007	3.501
Cytosine	1.415	0.501	0.001	0.013	3.152
1-Methyladenine	2.390	1.257	0.001	0.017	2.968
Uracil	1.870	0.903	0.001	0.017	2.899
Mandelic acid	1.378	0.463	0.003	0.034	2.542
L-cystathionine	0.187	-2.418	0.003	0.033	2.533
5-hydroxy-L-tryptophan	1.475	0.561	0.003	0.034	2.497
Malic acid	2.266	1.180	0.003	0.033	2.459

Metabolite	FC	$log_2(FC)$	p value	FDR-adjusted *p*-value	$-log_{10}(p)$
Hippuric acid	1.506	0.591	0.003	0.033	2.456
L-arginine	0.9185	-0.12265	0.004	0.038	2.3703
Thymidine	1.722	0.784	0.005	0.046	2.268
L-tyrosine	1.451	0.537	0.006	0.046	2.244
5-Formylcytosine	1.407	0.493	0.006	0.046	2.229
D-Glucuronic acid	1.533	0.616	0.008	0.056	2.108
Isovaleric acid	0.803	-0.316	0.008	0.058	2.073
1-Methyladenosine	2.447	1.291	0.010	0.062	2.015
Hydroxyphenylacetic acid	1.251	0.323	0.011	0.063	1.969
Phenylacetic acid	0.818	-0.289	0.011	0.063	1.962
Deoxyuridine	1.533	0.616	0.012	0.068	1.914
xanthine	1.377	0.461	0.013	0.071	1.890
5-Methylcytosine	1.376	0.461	0.015	0.077	1.818
Citric acid	2.165	1.114	0.020	0.085	1.709
5-hydroxyindoleacetic acid	1.854	0.891	0.021	0.089	1.681
L-cystine	0.497	-1.007	0.022	0.096	1.666

Metabolite	FC	$\log_2$(FC)	p value	FDR-adjusted *p*-value	$-\log_{10}$(p)
3-methylindole	0.716	-0.483	0.023	0.094	1.642
Syringic acid	1.573	0.653	0.025	0.100	1.598
3-methoxytyramine	1.307	0.386	0.026	0.100	1.593
trimethylamine	1.452	0.538	0.028	0.106	1.559
Sarcosine	0.653	-0.615	0.029	0.109	1.538
L-kynurenine	1.368	0.452	0.036	0.126	1.440
3-Phosphoglyceric acid	0.633	-0.660	0.038	0.128	1.422
4-Hydroxyproline	0.818	-0.290	0.040	0.133	1.397
4-hydroxybutyric acid	0.335	-1.576	0.042	0.139	1.373

Metabolite annotations are putative and were assigned based on one-dimensional ^{1}H-NMR chemical shifts, multiplicity patterns, and spectral matching against the Human Metabolome Database (HMDB). Annotations were not independently confirmed by two-dimensional NMR or LC–MS/MS analyses. False discovery rate (FDR)-adjusted p-values were calculated using the Benjamini–Hochberg procedure.

Supplementary Table S3 Sensitivity analysis restricted to non-drinkers comparing urinary metabolites between high- and low-$PM_{2.5}$ exposure groups.

Metabolite	*p*-value	FDR-adjusted *p*-value
**L-Tryptophan**	0.006	0.022
**Uracil**	0.004	0.022
**L-Cystathionine**	0.331	0.331
**Malic acid**	0.009	0.023
**Hippuric acid**	0.012	0.023
**L-Arginine**	0.143	0.190
**Xanthine**	0.059	0.094
**5-Hydroxyindoleacetic acid**	0.266	0.304

Sensitivity analysis was restricted to participants who reported no alcohol consumption. Group comparisons were performed using the Mann–Whitney U test. FDR-adjusted *p*-values were calculated using the Benjamini–Hochberg false discovery rate correction method.

Supplementary Table S4 Relative standard deviation (RSD) of metabolite peak intensities across pooled quality control (QC) samples.

Metabolite	Mean intensity	SD	RSD (%)
L-tryptophan	62.18	6.24	10.04
methylamine	28.08	6.231	22.19
Benzoic acid	161.81	56.01	34.62
2-Methylbutyrylglycine	9.17	2.96	32.33
2-methylhippuric acid	241.23	49.92	20.69
Cytidine	79.86	19.97	25.01
2-Fuoric acid	130.53	34.68	26.57
Cytosine	978.28	348.70	35.64
1-Methyladenine	311.18	41.23	13.25
Uracil	153.82	18.81	12.23
Mandelic acid	177.85	65.74	36.96
L-cystathionine	34.81	7.33	21.06
5-hydroxy-L-tryptophan	164.73	34.82	21.14
Malic acid	40.49	5.96	14.71
Hippuric acid	241.23	49.92	20.69
L-arginine	16.29	5.30	32.54
Thymidine	113.91	19.89	17.46
L-tyrosine	166.96	2.69	1.609
5-Formylcytosine	0.82	0.19	23.34
D-Glucuronic acid	104.88	26.15	24.94
Isovaleric acid	19.53	4.55	23.28
1-Methyladenosine	398.98	116.76	29.26
Hydroxyphenylacetic acid	82.89	24.55	29.62

Metabolite	Mean intensity	SD	RSD (%)
Phenylacetic acid	61.42	16.39	26.68
Deoxyuridine	123.93	29.96	24.17
Xanthine	202.49	8.86	4.37
5-Methylcytosine	398.63	95.72	24.01
Citric acid	19.605	5.84	29.81
5-hydroxyindoleacetic acid	57.38	8.92	15.55
L-cystine	55.54	20.64	37.16
3-methylindole	29.78	10.15	34.09
Syringic acid	133.26	30.56	22.93
3-methoxytyramine	107.70	36.83	34.19
trimethylamine	35.59	7.68	21.58
Sarcosine	33.77	11.19	33.12
L-kynurenine	196.09	77.25	39.39
3-Phosphoglyceric acid	61.43	19.79	32.21
4-Hydroxyproline	19.37	4.36	22.50
4-hydroxybutyric acid	59.74	16.74	28.01

RSD (%) was calculated as (standard deviation/mean intensity) × 100 across repeated pooled QC measurements.

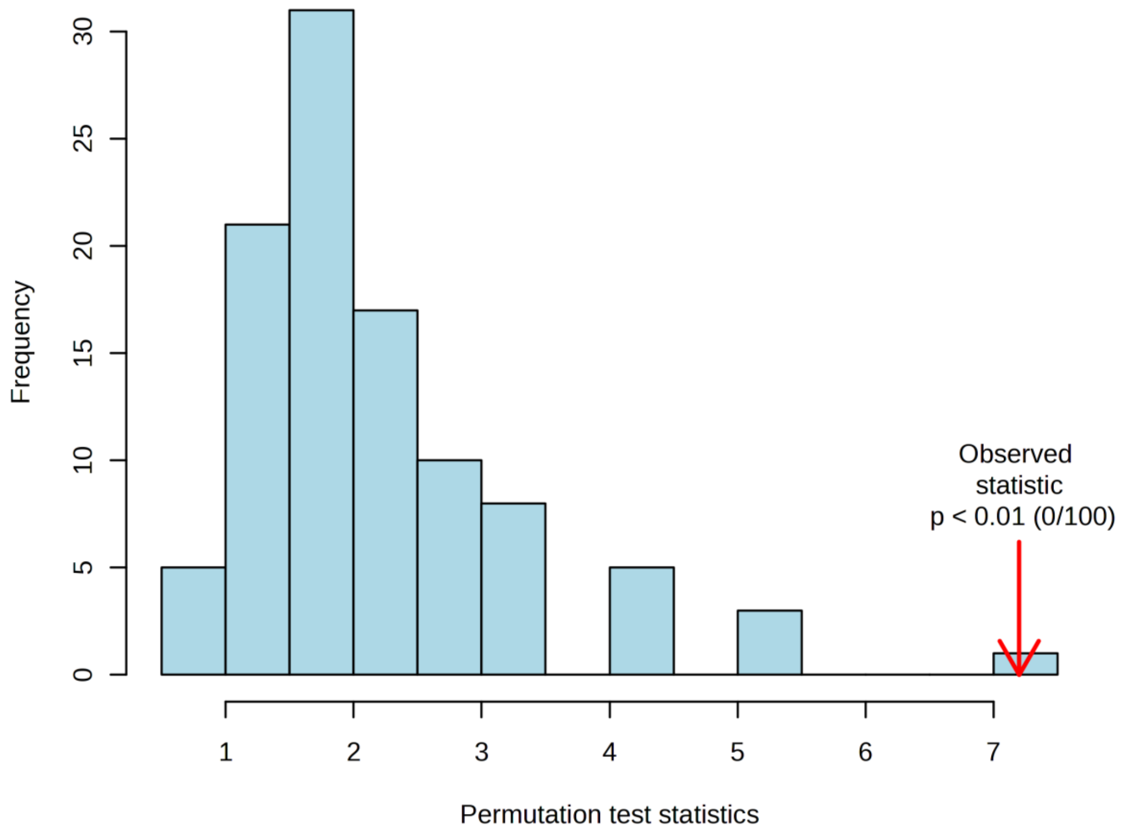

Supplementary Figure S1. Permutation test for PLS-DA model validation. The histogram shows the distribution of classification statistics obtained from 100 permuted models. The observed model (arrow) demonstrated significantly higher performance than permuted models ($p < 0.01$), indicating that the PLS-DA model was not overfitted.
